# *In silico* evaluation of gadofosveset pharmacokinetics in different population groups using the Simcyp® simulator platform

**DOI:** 10.1186/s40203-014-0002-x

**Published:** 2014-06-12

**Authors:** Marios Spanakis, Kostas Marias

**Affiliations:** Computational Medicine Laboratory, Institute of Computer Science, Foundation of Research & Technology-Hellas (FORTH), GR-71110 Heraklion, Crete Greece

**Keywords:** Gadofosveset, Gadolinium-based contrast agents, PBPK, Simcyp, DCE-MRI, Medical imaging, Pharmacokinetics

## Abstract

**Purpose:**

Gadofosveset is a Gd-based contrast agent used for magnetic resonance imaging (MRI). Gadolinium kinetic distribution models are implemented in T1-weighted dynamic contrast-enhanced perfusion MRI for characterization of lesion sites in the body. Physiology changes in a disease state potentially can influence the pharmacokinetics of drugs and to this respect modify the distribution properties of contrast agents. This work focuses on the *in silico* modelling of pharmacokinetic properties of gadofosveset in different population groups through the application of physiologically-based pharmacokinetic models (PBPK) embedded in Simcyp® population pharmacokinetics platform.

**Methods:**

Physicochemical and pharmacokinetic properties of gadofosveset were introduced into Simcyp® simulator platform and a min-PBPK model was applied. *In silico* clinical trials were generated simulating the administration of the recommended dose for the contrast agent (i.v., 30 mg/kg) in population cohorts of healthy volunteers, obese, renal and liver impairment, and in a generated virtual oncology population. Results were evaluated regarding basic pharmacokinetic parameters of Cmax, AUC and systemic CL and differences were assessed through ANOVA and estimation of ratio of geometric mean between healthy volunteers and the other population groups.

**Results:**

Simcyp® predicted a mean Cmax = 551.60 mg/l, a mean AUC = 4079.12 mg/L*h and a mean systemic CL = 0.56 L/h for the virtual population of healthy volunteers. Obese population showed a modulation in Cmax and CL, attributed to increased administered dose. In renal and liver impairment cohorts a significant modulation in Cmax, AUC and CL of gadofosveset is predicted. Oncology population exhibited statistical significant differences regarding AUC when compared with healthy volunteers.

**Conclusions:**

This work employed Simcyp® population pharmacokinetics platform in order to compute gadofosveset’s pharmacokinetic profiles through PBPK models and *in silico* clinical trials and evaluate possible differences between population groups. The approach showed promising results that could provide new insights regarding administration of contrast agents in special population cohorts. *In silico* pharmacokinetics could further be used for evaluating of possible toxicity, interpretation of MRI PK image maps and development of novel contrast agents.

## Background

**Figure 1:**
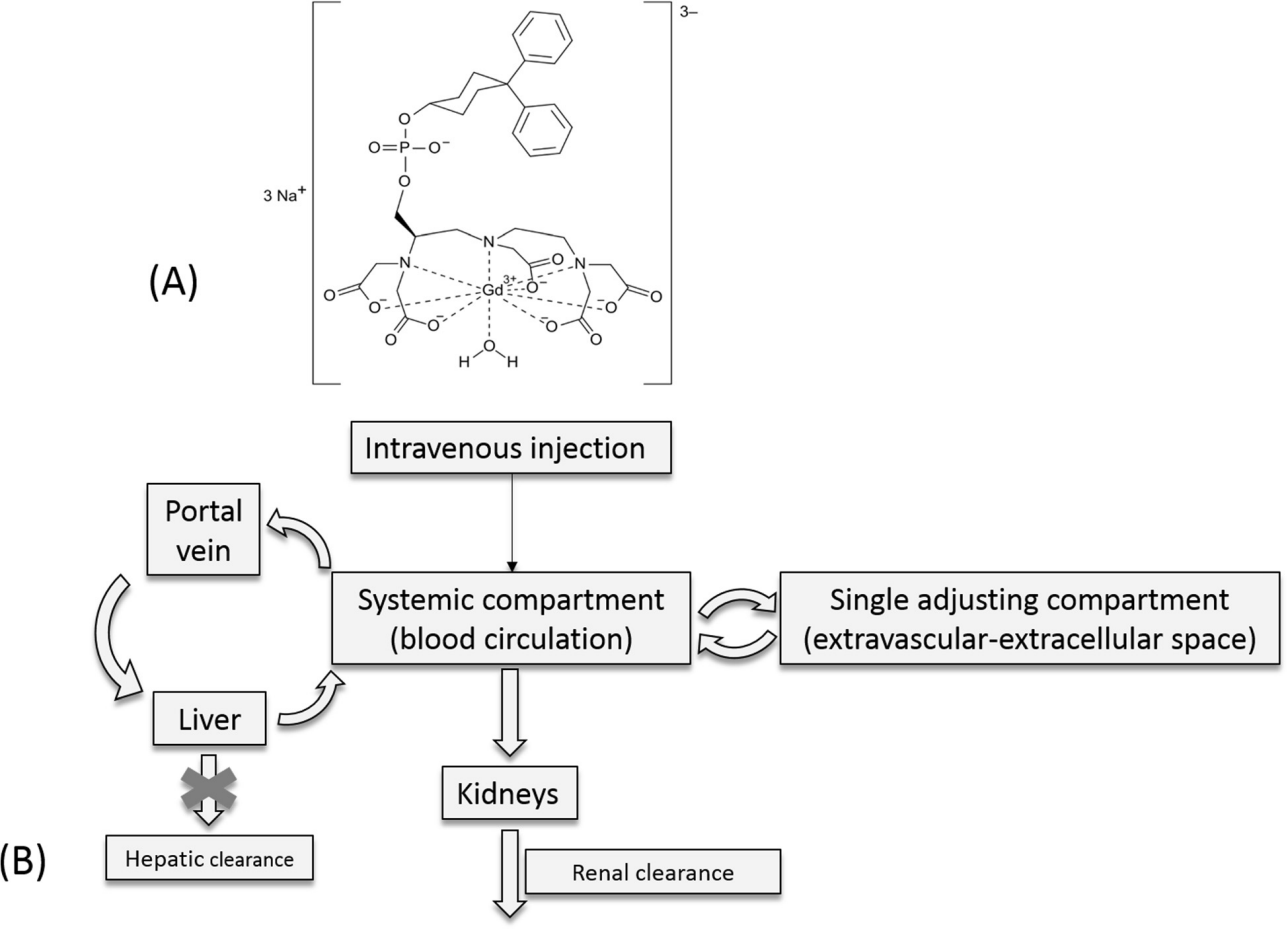
**Contrast agent and PBPK model used in this study. (A)** Chemical structure of gadofosveset. **(B)** Graphical representation of the min-PBPK model applied for gadofosveset from the Simcyp® simulator platform with hepatic clearance set to zero (x) and elimination occurring only from the systemic compartment through kidneys.

Gadofosveset trisodium (Vasovist®, Ablavar®, Figure 1A) is a Gd-based contrast agent (GBCA) used in dynamic-contrast enhancement magnetic resonance image (DCE-MRI). The mechanism of action of GBCAs in DCE-MRI relies in the alteration of relaxation times of atoms within body tissues due to the paramagnetic behavior of Gd and the interaction with nearby hydrogen nuclei which shortens the longitudinal relaxation (T1) times of water in the local tissue and increases signal intensity on T1-weighted images (Gossuin et al. 2010). In DCE-MRI, depending on the distribution rate of the contrast agent in a specific organ lesion, several essential information are gathered such as transfer constant rates (k^trans^, k_ep_), extravascular extracellular space volume per unit volume of tissue (v_e_), blood plasma volume per unit volume of tissue (v_p_) and the concentration-time profile in a near-by artery (arterial input function, AIF) (Koh et al. 2011; Tofts et al. 1999). A main characteristic of gadofosveset is the reversible binding to endogenous serum albumin with a moderate affinity (Kd = 85 μΜ) which leads in a prolonged vascular residence time compared to non-protein binding contrast agents and also facilitates high resolution in arterial and venus images (Caravan et al. 2002). Gadofosveset, as DCE-MRI contrast agent, has been applied for diagnosis and characterization of brain and rectal tumors associating DCE-MRI calculated parameters with microvascularity and in particular, with angiogenesis related leakage for tumorous areas (Lambregts et al. 2013; Puig et al. 2013). The contrast agent also belongs to the category of blood-pool contrast agents for magnetic resonance angiography (MRA) in cases of peripheral vascular disease (PVD) or aortoiliac occlusive disease (AIOD) (Goyen 2008). As a DCE-MRI contrast agent, gadofosveset is available in US with approval from FDA (FDA 2011), whereas in EU, the European Commission issued a decision (EMA/854517/2011) to withdraw the marketing authorization for gadofosveset based on commercial reasons from marketing authorization holder (MAH) (EMA 2011).

Regarding GBCA pharmacokinetics, after intravenous (i.v.) administration, GBCAs distribute in the blood and into extravascular-extracellular space. GBCAs follow a bi-compartment pharmacokinetic profile in the body with a distinct distribution and elimination phase (Aime and Caravan 2009). Gadofosveset, according to summary of product characteristics in humans, shows a mean distribution half-life of 0.48 ± 0.11 hours and a mean half-life of 16.3 ± 2.6 hours in elimination phase which is associated with albumin binding. The agent doesn’t follow any substantial biotransformation through metabolic processes and the volume of distribution is estimated approximately to be 148 ± 16 mL/kg. Gadofosveset is eliminated exclusively through kidneys in the urine with an estimated renal clearance of 6.57 ± 0.97 mL/h/kg. The 83.5% of an i.v. dose is excreted over 14 days and 94% of urinary excretion occurs during the first 72 hours. A small proportion of the dose is recovered in feces (Aime and Caravan 2009; FDA 2011).

Physiologically-based pharmacokinetic models (PBPK) represent a well-established approach in order to assess PK profiles of xenobiotics under various physiological conditions. PBPK models integrate data by taking into account drug-dependent and physiological related parameters (i.e. organ volume, demographics, disease, genetics etc.) as well as, their variation amongst individuals (Rowland et al. 2011). This approach allows the simulation and prediction of PK parameters of drugs in virtual populations and provides insights in several essential pharmacological questions such as PK profiles in special population groups (Atkinson and Smith 2012; Rostami-Hodjegan 2012). Simcyp® population-based simulator is a software for mechanistic PBPK modeling and simulation of pharmacokinetics and/or pharmacodynamics in virtual populations (http://www.simcyp.com). Apart of other advantages, the platform gives also the availability of conducting *in silico* clinical trials in different population groups based on disease state (Jamei et al. 2013).

Pharmacokinetic clinical data for administration of gadofosveset in special population groups are currently limited. Generally, renal impairment and in some cases liver deficiency, have been studied for possible modulation of GBCAs kinetics (Davies et al. 2002; Swan et al. 1999). Especially for renal impairment it is well-known that administration of GBCA for DCE-MRI is avoided due to the accumulation of Gd in the body and the high risk of presenting nephrogenic systemic fibrosis (NSF) (Abraham and Thakral 2008; Hasebroock and Serkova 2009; Grobner and Prischl 2007). Till today there are some case reports regarding NSF toxicity after administration of GBCAs in patients with several comorbidities and some considerations regarding possible toxicity in cancer patients (Gandhi et al. 2012; Launay-Vacher et al. 2007; Badero et al. 2008; Grebe et al. 2008). Furthermore, recently published works focus on physiology characteristics and their impact on estimation of DCE-MRI parameters (Just et al. 2011; Lavini and Verhoeff 2010). All the above are posing the question whether significant modulations of pharmacokinetics should be expected after administration of gadofosveset in special populations such as obese or cancer patients and also if they should be taken into consideration in the clinical level regarding toxicity or DCE-MRI parameter estimation.

The aim of this work was to assess, through PBPK models and *in silico* clinical trials, the PK profiles of gadofosveset in different populations in which the contrast agent could potentially be used for DCE-MRI studies and evaluate possible differences among these cohorts. To this respect, the use of *in silico* clinical trials approach was implemented through the Simcyp® population pharmacokinetics platform. Best to our knowledge for gadofosveset, this is the first attempt to calculate the PK profiles through the application of PBPK modeling and *in silico* clinical trials.

## Methods

Gadofosveset physicochemical and PK properties (Table 1) were obtained from Drugbank (Wishart et al. 2008) and were used to generate a compound in the Simcyp® simulator platform (Simcyp. V13 Simcyp Ltd, Sheffield, UK). In addition to these properties the albumin-binding (Kd = 85 μΜ) was introduced with simulator’s calculator to estimate that fraction unbound (fu) in plasma to be 0.11. Following the input of contrast-agent parameters, Simcyp’s min-PBPK model was applied in order to simulate the bi-compartmental behavior of gadofosveset. In this min-PBPK approach all organs and compartments (except liver and portal vein) are lumped and two more compartments are introduced, one representing the blood pool and a second, single adjusted compartment (Vsac), which in this case represented the extravascular-extracellular space (Figure 1B). GBCAs eliminate through kidneys and any modulation of kidney function is related with accumulation of Gd in the body and possible toxicity (Abraham and Thakral 2008; Amet and Deray 2012; Hasebroock and Serkova 2009). To this respect, elimination settings for organ metabolic clearance adjusted to zero for all organs and elimination set to be occurred exclusively from the systemic compartment through kidneys (Figure 1B). In addition, following the reported value of clearance for gadofosveset, a typical value of renal clearance for a healthy male 20–30 years old set to be 0.5 L/h. Through this approach, simulator set to estimate through its algorithms, gadovosveset’s clearance in all population groups based in modulation of renal function due to the disease or physiology changes taking into account the clearance value of a healthy male 20–30 years old.

**Table 1:** **Basic physicochemical and pharmacokinetic properties of gadofosveset that were used in the Simcyp® simulator platform**

**Physicochemical properties**	
Molecular Weight (g/mol)	975.87 g/mol
pKa (acid)	0.78
pKa (base)	9.67
logP	-1.2
PSA	268.96
**Pharmacokinetic properties**	
Dose (IV)	0.03 mmol/Kg (or 30 mg/kg)
CL (mL/min/kg)	6.57 ± 0.97 ml/h/kg
Vd L/Kg	0.15 ± 0.01 ml/Kg (fu = 15–20%)
Elimination t1/2	18.5 h
Distribution t1/2	0.48 h
Route of elimination	Kidneys (94% of urinary excretion occurs in the first 72 hr)

The simulated clinical trials carried out in the following virtual population groups of Simcyp’s platform: i) healthy volunteers, ii) renal impairment with GFR values between 30–60 iii) renal impairment with GFR below 30, iv) obese, and v) Liver cirrhosis (types A, B, C). Also *in silico* clinical trials were conducted in a virtual oncology group generated according to a recently published work (Cheeti et al. 2013). All simulations run for 10 clinical trials of 10 subjects in each trial (0.5 females). In order to assess the prolonged elimination of gadofosveset, the *in silico* clinical trials were generated over a time period of 72 hours following the usually administered i.v. dose of the contrast agent (30 mg/kg).

The obtained results were evaluated in GraphPad Prism® (v5.01 GraphPad Software Inc.) for possible statistical significant differences through ANOVA using Dunnet’s test in order to compare all populations with healthy volunteers, (95% confidence intervals) following log-transformation. The ratio of geometric mean (GMR ± 0.2) for Cmax, AUC and CL between healthy volunteers (control group) and other population groups was used to briefly estimate the equivalence of gadofosvesest administration between population cohorts.

## Results and discussion

**Figure 2:**
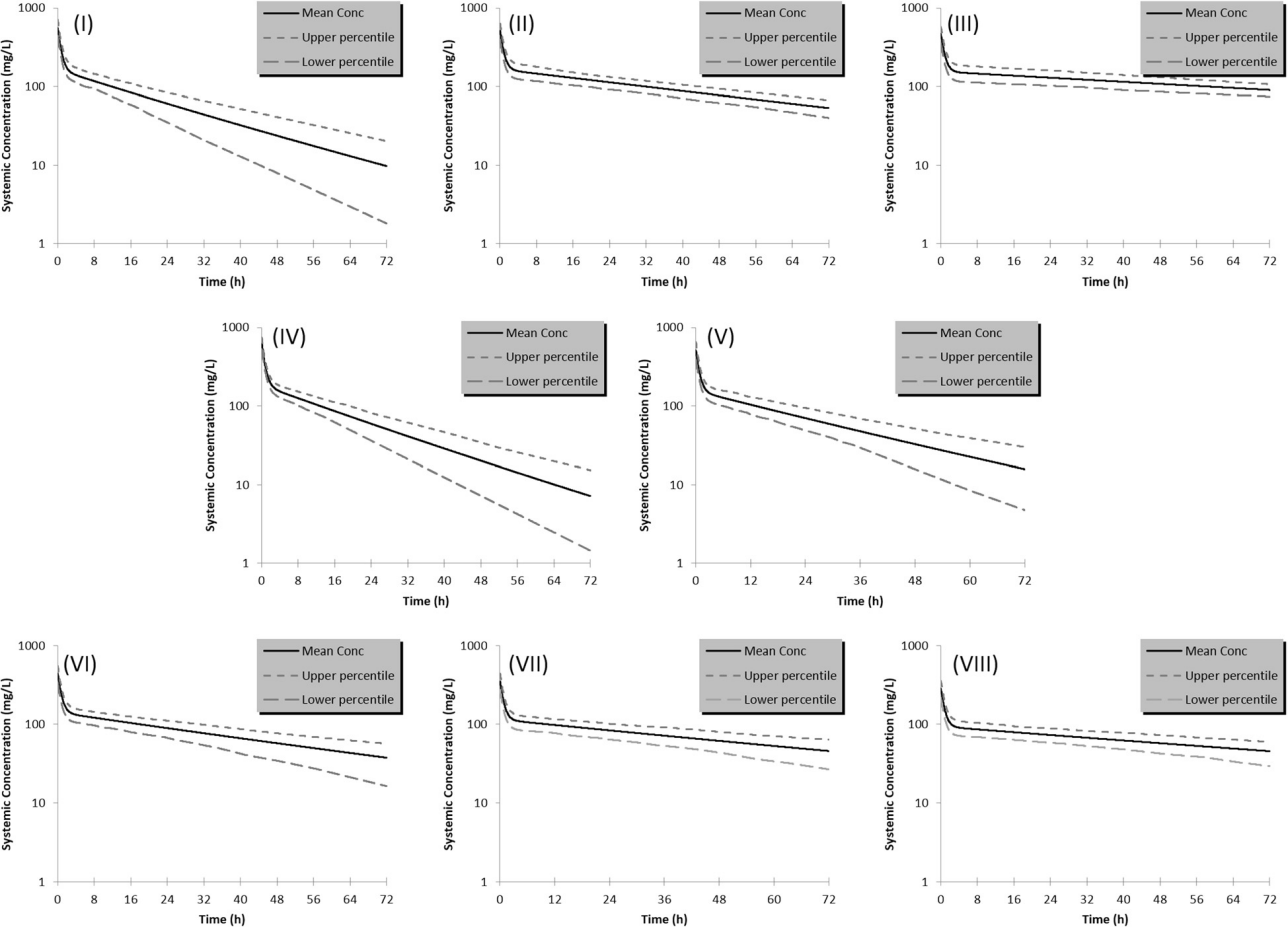
**Semi-log plots of systemic concentration in plasma over time of gadofosveset in the simulated populations groups. (I)** Healthy volunteers, **(II)** Renal impairment (GFR30-60) **(III)** Renal impairment (GFR < 30), **(IV)** Obese, **(V)** Oncology, **(VI)** Liver cirrhosis type A, **(VII)** Liver cirrhosis type B, **(VIII)** Liver cirrhosis type C.

**Figure 3:**
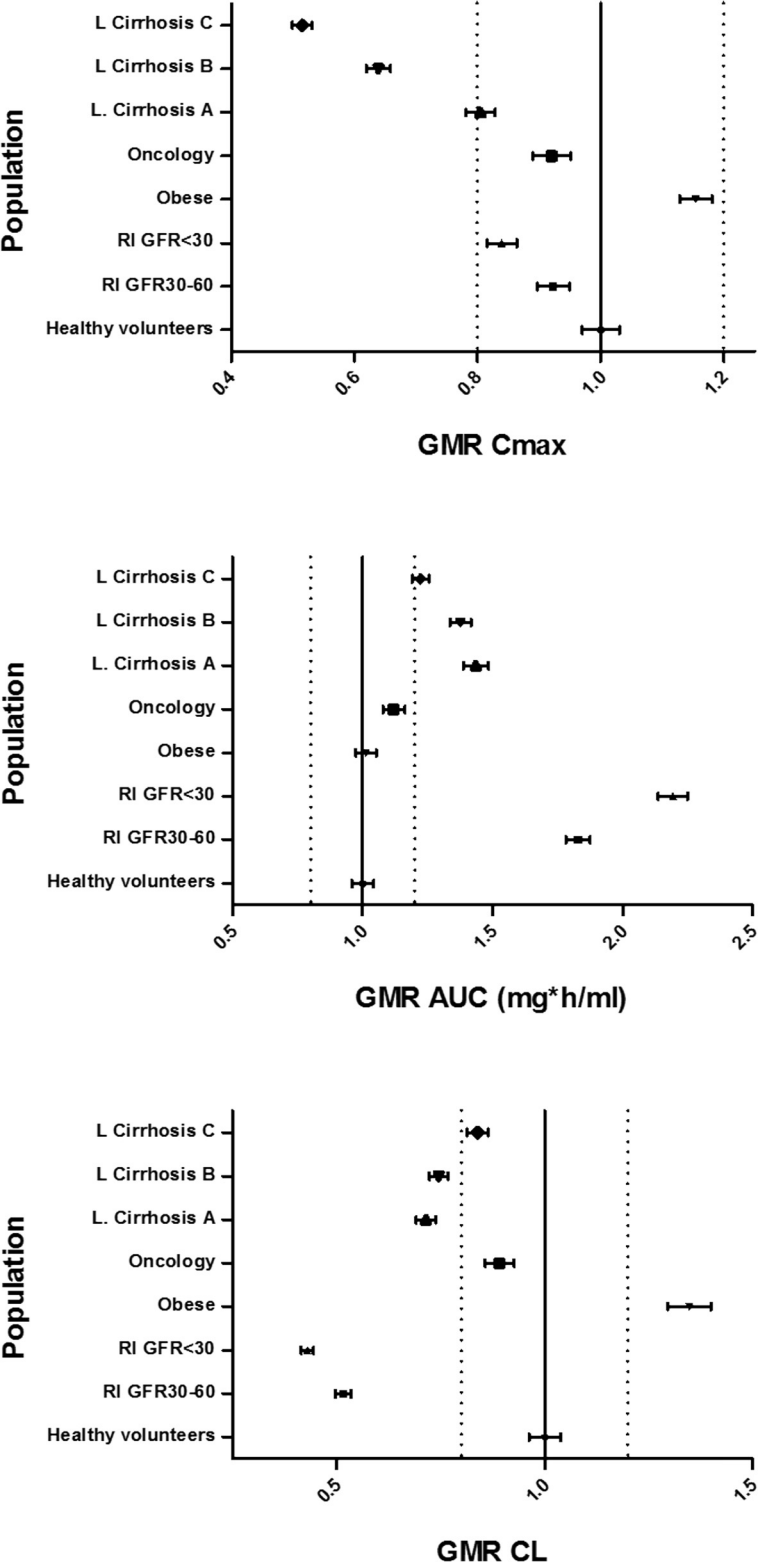
**Geometric mean ratio (GMR ± 95% upper or lower limit) of Cmax, AUC amd CL values for gadofosveset in the simulated population groups.** Dashed vertical lines represent values of 0.8 and 1.2 below and above GMR between healthy volunteers and the other simulated populations.

**Figure 4:**
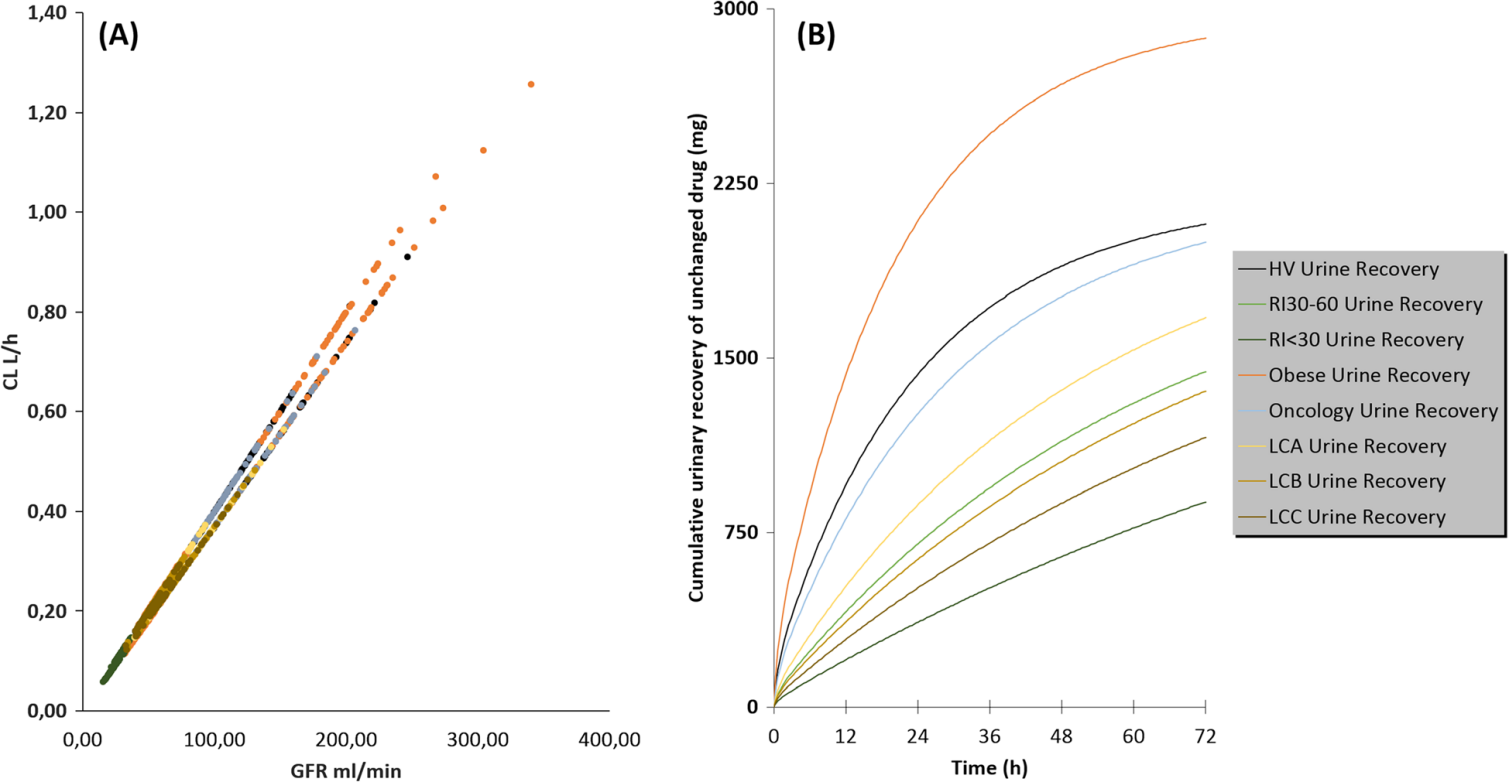
**Renal elimination and urinary recovery of gadofosveset. (A)** Linear correlation of GFR and systemic clearance of gadofosveset in simulated individuals in all population groups. **(B)** Calculated mean urinary recovey of gadofosveset in different population groups over a time period of 72 h.

The calculated pharmacokinetic parameters of gadofosveset are presented in Table 2 and the concentration-time profiles of the mean values along with the upper and lower percentile for each population are illustrated in Figure 2(I-VIII). Pharmacokinetic profiles and parameters seem to differentiate in the other population cohorts as it is shown in Figure 2(II-VIII) and Table 2. Figure 3 shows the modulation from the ratio of geometric mean of Cmax, AUC and CL between healthy volunteers and the other population groups. The elimination of gadofosveset was in linear correlation to kidney function and GFR for all population groups and the cumulative amount –or the fraction of administered dose – of contrast agent excreted in urine was similar in most cases except in kidney and liver impairment (Table 2, Figure 4).

**Table 2:** Mean administered doses (total mg) and mean (±SD) predicted values of Cmax (mg/L), AUC (mg*L/h), systemic CL (L/h) and fraction of dose excreted in urine (fe) for gadofosveset in simulated populations

	**Pharmacokinetic parameter**				
**Population group**	**Dose (mg)**	**Cmax (mg/L)**	AUC (mg/L*h)	**CL(L/h)**	**fe**
Healthy volunteers	2212.90 (±385.49)	551,60 (±84.29)	4079.12 (±822.39)	0,56 (±0.11)	0.94 (±0.04)
Renal impairment GFR 30–60	2104.51 (±453.87)	508.34 (±73.37)	7364.89 (±931.76)***	0.29 (±0.05)***	0.68 (±0.05)***
Renal impairment GFR < 30	2104.51 (±453.87)	462.46 (±65.38)***	8837.03 (±1135.09)***	0.24 (±0.04)***	0.42 (±0.05)***
Obese	2997.52 (±373.76)***	633.54 (±72.04)***	4122.79 (±769.33)	0.75 (±0.11)***	0.96 (±0.04)
Oncology	2228.18 (±478.72)	509.27 (±87.81)	4551.60 (±841.70)*	0.50 (±0.09)	0.90 (±0.06)
Liver cirrhosis type A	2283.12 (±459.26)	443.61 (±65.99)***	5814.62 (±942.87)***	0.40 (±0.06)***	0.74 (±0.03)***
Liver cirrhosis type B	2283.12 (±459.26)	352.75 (±55.22)***	5557.40 (±798.26)***	0.41 (±0.06)***	0.60 (±0.11)***
Liver cirrhosis type C	2283.12 (±459.26)	284.60 (±44.89)***	4932.06 (±668.71)***	0.46 (±0.07)**	0.51 (±0.11)***

*P < 0.05, **P < 0.01, ***P < 0.001 statistical significant differences with control group (healthy volunteers).

The results from population of healthy volunteers predict a mean Cmax = 551.60 mg/L, a mean AUC = 4079.12 mg*h/L and a systemic CL of 0.56 L/h (or 7.56 L/h/kg) for gadofosveset. Moreover the mean fraction of dose eliminated through kidneys (fe) over a time period of 72 predicted to be 0.94 (Table 2). The calculated values for virtual population of healthy volunteers, mainly CL and fe, are in good correlation with values from the literature and are presented in Table 3 (FDA 2011; Wishart et al. 2008). Theoretically, taking into account the protein binding, renal elimination of gadofosveset expected to be 0.86 L/h (fu*GFR) whereas the predicted (0.56 L/h) for healthy volunteers) as well as the reported (0.49 L/h) values of renal elimination are much lower. This finding suggests a possible active tubular reabsorption of the contrast agent but till today there are no any data available for active transport during elimination and thus far no transporters have been identified where the agent could serve as substrate. The only contrast agents with active transport are gadobenate dimeglumin (Gd-BOPTA, MultiHance®) and gadoxetic acid (Gd-EOB-DTPA, Primovist®) (Pascolo et al. 1999). As a result, due to the good correlation of *in silico* data with reported values (Table 3) this approach through Simcyp® seems capable to predict the basic pharmacokinetic profile and parameters of gadofosveset compared with reported values and as a result, healthy volunteers served as control group towards the evaluation of the PK parameters in the other population cohorts.

**Table 3:** **Comparison of the pharmacokinetic parameters that were predicted through Simcyp® simulator with the values available from the literature (FDA and Drugbank) for populations of healthy volunteers and renal impairment patients**

**PK Parameter**	**Predicted from Simcyp®**	**Data from FDA/Drugbank**
C_max_ (mg/L) (mean ± SD)	551.60 (±84,29)	419.6 (±39.04)
C _t=1h_ (mg/L) (mean ± SD)	256.17 (±35.41)	234.21 (±29.27)
fu	0.11	0.12–0.20
CL (mL/h/kg) (mean ± SD)	7.56 (±1.46)	6.57 (±0.97)
fe (72 h)	0.94	0.94
AUC fold increase in moderate renal impairment	1.8	1.75
AUC fold increase in severe renal impairment	2.2	2.25

In the two renal impairment populations, the simulations successfully predicted the expected and statistically significant delayed elimination and accumulation in the body of gadofosveset compared to the healthy volunteers (P < 0.001) (Figure 2I-III). The results from Simcyp® for administration of gadofosveset in renal impairment predict a slight modulation in Cmax whereas a 1.8 and 2.2 fold increase in AUC with the respective decrease in CL was predicted for the two cohorts of renal deficiency (Figure 3) which were in good correlation with known data (Table 3). The accumulation of gadofosveset in the body was also associated with disease severity and the decrease in GFR values (Figures 2II, III and 4). These results seem to correlate with the known impact of kidney function in GBCA elimination and increased risk of toxicity and potentially could be used to further explain cases of acute renal failure and Gd-toxicity (Bhaskaran et al. 2010).

For obese population (Figure 2IV), a statistically significant increase in Cmax and CL (P < 0.001) was predicted without modulation in calculated AUC (Figure 3). This difference can be attributed to the higher total administered dose of the contrast-agent based on the body weight (Table 2). The fraction of the dose eliminated in obese people seems to remain similar (fe = 0.96) with healthy volunteers of normal body weight but the increased total administered dose leads in a higher cumulative amount of gadofosveset that is calculated to be excreted in the urine (Table 2, Figure 4B). Obese population was applied due to the administration of GBCA based on body weight and in an effort to assess the possible impact on gadofosveset pharmacokinetic parameters. Previously published studies with PBPK models have shown that for several pharmacokinetic parameters, especially for clearance, variations due to increased body weight should be expected (Ghobadi et al. 2011). The increased exposure in gadolinium potentially could be related with toxicity and also regarding the DCE-MRI could lead in an increased signal intensity.

Regarding the oncology group, a statistical significant difference in AUC is estimated (P < 0.05) whereas modulation in Cmax and CL is not predicted compared with the population of healthy volunteers (Figure 3 and Table 2). The generated oncology population was based on general physiology changes that are observed in cancer patients without taking into account possible organ dysfunction, especially in kidneys, with the potential impact on contrast agent PKs and moreover in presenting adverse reactions (Amet and Deray 2012; Launay-Vacher et al. 2007). In addition, although the clinical impact in DCE-MRI of the modulation of AUC between healthy volunteers and cancer patients remains to be further addressed, it is expected to contribute in the interpretation of the imaging results (e.g. role of PK in tumor characterization).

Populations of liver cirrhosis (types A, B, C), exhibited a decreased Cmax concentration compared with other groups and a similar trend in c-t profiles with those of renal impairment cohorts (Figure 2VI-VIII, Table 2). In addition, results from simulations calculate a statistical significant increase in AUC and decrease in CL in these cohorts. Furthermore, modulation in Cmax, AUC and CL seem to follow disease severity and progression (Figure 3). These findings could be attributed with the changes in physiology observed and taken into consideration during simulations in liver deficiency that lead in decreased renal function and hepatorenal syndrome (Lata 2012). As a result, administration of gadofosveset for DCE-MRI in patients with liver cirrhosis could take into consideration the possible hepatorenal syndrome in order to prevent potential toxicity. Also a modulation from the expected values, mainly in AIF, could be expected in DCE-MRI setting. Comparing these findings with FDA’s SPC though, pharmacokinetics and plasma protein binding of gadofosveset have not reported till today to be significantly influenced by moderate hepatic impairment and the only value that is influenced is the fecal elimination of the contrast agent which is reduced in hepatic impaired subjects (FDA 2011).

Summarizing the above observations, application of Simcyp® simulator platform on gadofosveset and the incorporation of *in silico* clinical trials shows potential in estimating the pharmacokinetic properties and profiles of gadofosveset and identify possible differences between several population groups. PBPK models and simulators for *in silico* clinical trials such as Simcyp® can be promising in providing new insights regarding the pharmacokinetic behavior of contrast agents in the body and the variability in the estimated MRI values, especially in disease population groups where clinical trials cannot easily been conducted. Thus far, Simcyp® simulator platform has been applied in predicting PK profiles of drugs in special population groups such as obese, renal deficiency, liver impairment and rheumatoid arthritis with good correlation between disease model and clinical data (Johnson et al. 2010; Machavaram et al. 2013; Rowland Yeo et al. 2011; Ghobadi et al. 2011). Although, Simcyp® platform represents a “bottom-up” PBPK approach where *in vitro* data are extrapolated to possible *in vivo* results, in this study, the *in silico* clinical trials implementations were based on known *in vivo* parameters in an effort to combine clinical data to “bottom-up” PBPK models (Tsamandouras et al. 2013). The application of PBPK models and the results from *in silico* clinical trials can also be applied towards the development of novel GBCAs or for further developing/improving models for MRI PK analysis (Huang and Tsourkas 2013; Lim et al. 2012; Brochot et al. 2006; Bui et al. 2010).

Overall, the proposed application of *in silico* clinical trials for gadofosveset, represent a novel approach for the estimation of PK parameters and population variability regarding GBCAs. The observations from the *in silico* population analysis revealed several new aspects that can possibly be evaluated with clinical observations for gadofosveset which till today are limited. Moreover, PBPK models can provide tools where differences in acquired MRI images could be attributed in physiology characteristics and not strictly to an observed lesion which potentially would provide new insights for MRI image analysis regarding GBCAs pharmacokinetics modeling. The exploitation of the results and correlation with clinical findings, along with systems biology tools and interfaces integrating patients profiles could further empower decision making tools in predicting the Gd concentration variability in patients and therefore assisting the clinician to better explain interesting findings in PK-derived disease related biomarkers (Spanakis et al. 2013).

## Conclusion

The PBPK-based *in silico* analysis results showed a good correlation with the literature related to gadofosveset’s pharmacokinetic parameters. Differences between healthy volunteers and specific population groups were observed and discussed regarding the potential impact in DCE-MRI and toxicity. Prospectively, this approach based on *in silico* populations has the potential to shed light in the understanding MRI PK parameters variability observed in clinical practice, lead to more robust MRI biomarkers by factoring out population-dependent PK variability and enhance drug development processes for novel contrast agents. Towards this goal, we aim to extend our work in coupling the application of PBPK models with *in silico* clinical trials for optimizing the clinical value of MRI biomarkers.

## Abbreviations

GBCA: Gd-Based contrast agents
PK: Pharmacokinetics
DCE-MRI: Dynamic contrast enhancement magnetic resonance imaging
PBPK: Physiologically-based pharmacokinetic models
Cmax: Maximum concentration in plasma
AUC: Area Under the Curve in concentration-time plots
CL: Clearance
AIF: Arterial Input Function

## References

[CR1] Abraham JL, Thakral C (2008). Tissue distribution and kinetics of gadolinium and nephrogenic systemic fibrosis. Eur J Radiol.

[CR2] Aime S, Caravan P (2009). Biodistribution of gadolinium-based contrast agents, including gadolinium deposition. J Magn Reson Imaging.

[CR3] Amet S, Deray G (2012). Renal toxicity of contrast agents in oncologic patients. Bull Cancer.

[CR4] Atkinson AJ, Smith BP (2012). Models of physiology and physiologically based models in clinical pharmacology. Clin Pharmacol Ther.

[CR5] Badero OJ, Schlanger L, Rizk D (2008). Gadolinium nephrotoxicity: case report of a rare entity and review of the literature. Clin Nephrol.

[CR6] Bhaskaran A, Kashyap P, Kelly B, Ghera P (2010). Nephrogenic systemic fibrosis following acute kidney injury and exposure to gadolinium. Indian J Med Sci.

[CR7] Brochot C, Bessoud B, Balvay D, Cuenod CA, Siauve N, Bois FY (2006). Evaluation of antiangiogenic treatment effects on tumors’ microcirculation by Bayesian physiological pharmacokinetic modeling and magnetic resonance imaging. Magn Reson Imaging.

[CR8] Bui T, Stevenson J, Hoekman J, Zhang S, Maravilla K, Ho RJ (2010) Novel Gd nanoparticles enhance vascular contrast for high-resolution magnetic resonance imaging. PLoS One 5(9), doi:10.1371/journal.pone.0013082 10.1371/journal.pone.0013082 PMC2948015 20927340

[CR9] Caravan P, Cloutier NJ, Greenfield MT, McDermid SA, Dunham SU, Bulte JW, Amedio JC, Looby RJ, Supkowski RM, Horrocks WD, McMurry TJ, Lauffer RB (2002). The interaction of MS-325 with human serum albumin and its effect on proton relaxation rates. J Am Chem Soc.

[CR10] Cheeti S, Budha NR, Rajan S, Dresser MJ, Jin JY (2013). A physiologically based pharmacokinetic (PBPK) approach to evaluate pharmacokinetics in patients with cancer. Biopharm Drug Dispos.

[CR11] Davies BE, Kirchin MA, Bensel K, Lorusso V, Davies A, Parker JR, Lafrance ND (2002). Pharmacokinetics and safety of gadobenate dimeglumine (multihance) in subjects with impaired liver function. Invest Radiol.

[CR12] EMA (2011). Public statement on: Ablavar (gadofosveset) withdrawal of the marketing authorisation in the European Union European Medicines Agency.

[CR13] FDA (2011). Ablavar (gadofosveset trisodium) prescribing information December 2010.

[CR14] Gandhi A, Moorthy B, Ghose R (2012). Drug disposition in pathophysiological conditions. Curr Drug Metab.

[CR15] Ghobadi C, Johnson TN, Aarabi M, Almond LM, Allabi AC, Rowland-Yeo K, Jamei M, Rostami-Hodjegan A (2011). Application of a systems approach to the bottom-up assessment of pharmacokinetics in obese patients: expected variations in clearance. Clin Pharmacokinet.

[CR16] Gossuin Y, Hocq A, Gillis P, Vuong QL (2010). Physics of magnetic resonance imaging: from spin to pixel. J Phys D Appl Phys.

[CR17] Goyen M (2008). Gadofosveset-enhanced magnetic resonance angiography. Vasc Health Risk Manag.

[CR18] Grebe SO, Borrmann M, Altenburg A, Wesselman U, Hein D, Haage P (2008). Chronic inflammation and accelerated atherosclerosis as important cofactors in nephrogenic systemic fibrosis following intravenous gadolinium exposure. Clin Exp Nephrol.

[CR19] Grobner T, Prischl FC (2007). Gadolinium and nephrogenic systemic fibrosis. Kidney Int.

[CR20] Hasebroock KM, Serkova NJ (2009). Toxicity of MRI and CT contrast agents. Expet Opin Drug Metabol Toxicol.

[CR21] Huang CH, Tsourkas A (2013). Gd-based macromolecules and nanoparticles as magnetic resonance contrast agents for molecular imaging. Curr Top Med Chem.

[CR22] Jamei M, Marciniak S, Edwards D, Wragg K, Feng K, Barnett A, Rostami-Hodjegan A (2013). The simcyp population based simulator: architecture, implementation, and quality assurance. In Silico Pharmacol.

[CR23] Johnson TN, Boussery K, Rowland-Yeo K, Tucker GT, Rostami-Hodjegan A (2010). A semi-mechanistic model to predict the effects of liver cirrhosis on drug clearance. Clin Pharmacokinet.

[CR24] Just N, Koh DM, D’Arcy J, Collins DJ, Leach MO (2011). Assessment of the effect of haematocrit-dependent arterial input functions on the accuracy of pharmacokinetic parameters in dynamic contrast-enhanced MRI. NMR Biomed.

[CR25] Koh TS, Bisdas S, Koh DM, Thng CH (2011). Fundamentals of tracer kinetics for dynamic contrast-enhanced MRI. J Magn Reson Imaging.

[CR26] Lambregts DM, Heijnen LA, Maas M, Rutten IJ, Martens MH, Backes WH, Riedl RG, Bakers FC, Cappendijk VC, Beets GL, Beets-Tan RG (2013). Gadofosveset-enhanced MRI for the assessment of rectal cancer lymph nodes: predictive criteria. Abdom Imaging.

[CR27] Lata J (2012). Hepatorenal syndrome. World J Gastroenterol.

[CR28] Launay-Vacher V, Oudard S, Janus N, Gligorov J, Pourrat X, Rixe O, Morere JF, Beuzeboc P, Deray G, Renal I, Cancer Medications Study G (2007). Prevalence of Renal Insufficiency in cancer patients and implications for anticancer drug management: the renal insufficiency and anticancer medications (IRMA) study. Cancer.

[CR29] Lavini C, Verhoeff JJ (2010). Reproducibility of the gadolinium concentration measurements and of the fitting parameters of the vascular input function in the superior sagittal sinus in a patient population. Magn Reson Imaging.

[CR30] Lim J, Turkbey B, Bernardo M, Bryant LH, Garzoni M, Pavan GM, Nakajima T, Choyke PL, Simanek EE, Kobayashi H (2012). Gadolinium MRI contrast agents based on triazine dendrimers: relaxivity and *in vivo* pharmacokinetics. Bioconjug Chem.

[CR31] Machavaram KK, Almond LM, Rostami-Hodjegan A, Gardner I, Jamei M, Tay S, Wong S, Joshi A, Kenny JR (2013). A physiologically based pharmacokinetic modeling approach to predict disease-drug interactions: suppression of CYP3A by IL-6. Clin Pharmacol Ther.

[CR32] Pascolo L, Cupelli F, Anelli PL, Lorusso V, Visigalli M, Uggeri F, Tiribelli C (1999). Molecular mechanisms for the hepatic uptake of magnetic resonance imaging contrast agents. Biochem Biophys Res Commun.

[CR33] Puig J, Blasco G, Essig M, Daunis IEJ, Laguillo G, Quiles AM, Remollo S, Bergmann K, Joly C, Bernado L, Sanchez-Gonzalez J, Pedraza S (2013). Albumin-binding MR blood pool contrast agent improves diagnostic performance in human brain tumour: comparison of two contrast agents for glioblastoma. Eur Radiol.

[CR34] Rostami-Hodjegan A (2012). Physiologically based pharmacokinetics joined with *in vitro*-*in vivo* extrapolation of ADME: a marriage under the arch of systems pharmacology. Clin Pharmacol Ther.

[CR35] Rowland Yeo K, Aarabi M, Jamei M, Rostami-Hodjegan A (2011). Modeling and predicting drug pharmacokinetics in patients with renal impairment. Expert Rev Clin Pharmacol.

[CR36] Rowland M, Peck C, Tucker G (2011). Physiologically-based pharmacokinetics in drug development and regulatory science. Annu Rev Pharmacol Toxicol.

[CR37] Spanakis M, Papadaki E, Kafetzopoulos D, Karantanas A, Maris TG, Sakkalis V, Marias K (2013). Exploitation of patient avatars towards stratified medicine through the development of in silico clinical trials approaches. Bioinformatics and Bioengineering (BIBE), 2013 IEEE 13th international conference on, 10-13 Nov. 2013.

[CR38] Swan SK, Baker JF, Free R, Tucker RM, Barron B, Barr R, Seltzer S, Gazelle GS, Maravilla KR, Barr W, Stevens GR, Lambrecht LJ, Pierro JA (1999). Pharmacokinetics, safety, and tolerability of gadoversetamide injection (OptiMARK) in subjects with central nervous system or liver pathology and varying degrees of renal function. J Magn Reson Imaging.

[CR39] Tofts PS, Brix G, Buckley DL, Evelhoch JL, Henderson E, Knopp MV, Larsson HB, Lee TY, Mayr NA, Parker GJ, Port RE, Taylor J, Weisskoff RM (1999). Estimating kinetic parameters from dynamic contrast-enhanced T(1)-weighted MRI of a diffusable tracer: standardized quantities and symbols. J Magn Reson Imaging.

[CR40] Tsamandouras N, Rostami-Hodjegan A, Aarons L (2013) Combining the “bottom-up” and “top-down” approaches in pharmacokinetic modelling: fitting PBPK models to observed clinical data. Br J Clin Pharmacol, doi:10.1111/bcp.12234 10.1111/bcp.12234 PMC4294076 24033787

[CR41] Wishart DS, Knox C, Guo AC, Cheng D, Shrivastava S, Tzur D, Gautam B, Hassanali M (2008). DrugBank: a knowledgebase for drugs, drug actions and drug targets. Nucleic Acids Res.

